# Incidence and risk factors for heat-related illness (heatstroke) in UK dogs under primary veterinary care in 2016

**DOI:** 10.1038/s41598-020-66015-8

**Published:** 2020-06-18

**Authors:** Emily J. Hall, Anne J. Carter, Dan G. O’Neill

**Affiliations:** 1 School of Animal, Rural and Environmental Sciences, Nottingham Trent University, Brackenhurst, Southwell, Notts NG25 0QF UK; 2 Pathobiology and Population Sciences, The Royal Veterinary College, Hawkshead Lane, North Mymms, Hatfield, Herts AL9 7TA UK

**Keywords:** Risk factors, Diseases

## Abstract

As climate change causes global temperatures to rise, heat-related illness, a potentially fatal condition in dogs, will become an ever-greater threat. This study aimed to report the incidence, fatality and canine risk factors of heat-related illness in UK dogs under primary veterinary care in 2016. The VetCompass^TM^ programme collects de-identified electronic patient records from UK veterinary practices for research. From the clinical records of 905,543 dogs under veterinary care in 2016, 395 confirmed heat-related illness events were identified. The estimated 2016 incidence of heat-related illness was 0.04% (95% CI 0.04-0.05%), with an event fatality rate of 14.18% (95% CI 11.08 – 17.96%). Multivariable analysis identified significant risk factors including breed (e.g. Chow Chow, Bulldog and French Bulldog), higher bodyweight relative to the breed/sex mean and being over two years of age. Dogs with a brachycephalic skull shape and dogs weighing over 50 kg were also at greater risk. As we move into an ever-warmer world, veterinary professionals may need to include resistance to heat-related illness amongst their rationales when advising owners on breed selection. Breeding for good respiratory function and maintaining a healthy bodyweight should be considered key welfare priorities for all dogs to limit the risk of heat-related illness.

## Introduction

Climate change is listed among the World Health Organisation’s top ten threats to Global Health in 2019, with heat-related illness (HRI) predicted to contribute towards an additional 250,000 human deaths annually by 2030^1^. HRI is a progressive disorder of animals and man caused by core body temperatures that rise above homeostatic limits, resulting in metabolic disturbances^2^. This can lead to decreased cardiac output, fatigue of heat dissipation mechanisms, organ failure and ultimately death^2–4^. Animal welfare organisations in the United Kingdom (UK) and Australia have reported increasing numbers of calls about animals trapped in hot environments over recent years^5,6^. As the frequency and severity of heat waves is predicted to increase, there is an urgent need for better evidence-based guidance on the risk factors and early recognition of HRI to improve prevention and treatment strategies for both humans and animals^3,7^.

A deficiency of reliable and current data on the diagnosis, treatment and fatality rate of HRI is a key barrier to mitigating HRI risks in both humans and dogs^8^. The classical terminology used to define HRI varies, but typically includes terms that describe progression from heat stress, through heat exhaustion to heatstroke^2^. However, these classical terms lack clear explicit definitions and are often used interchangeably as synonyms, leaving their usage open to individual interpretation that creates a confused medical and veterinary literature^3^. A novel HRI scoring system has been proposed for use in humans, acknowledging that patients can progress through the stages of disease severity depending on the duration and intensity of heat exposure and effectiveness of treatment^9^. To date, studies of HRI in dogs have included only cases described as suffering from advanced stages of HRI “heatstroke”^10–13^. Excluding dogs presenting with less severe forms and stages of HRI from risk factor analysis fails to take the progressive nature of the condition into consideration and biases the results away from the overall HRI caseloads seen in general veterinary practice.

HRI is reported as a relatively common condition in dogs in regions with hot climates^3,12^, but cases are reportedly less common in more temperate regions such as the UK. In a BVA survey of over 1000 UK companion animal veterinarians, half reported seeing an average of five canine heat-related illness cases during the summer of 2016^14^. Case reviews from primary-care single centre studies in the UK often include insufficient cases for robust statistical analyses and are therefore of limited scientific value. For this reason, canine HRI studies to date have tended to rely on referral hospital populations that accumulate caseloads from a broad base of referring practices^10–13,15,16^, but referral caseloads self-select for complex and severe cases, and the diagnoses and outcomes will be heavily influenced by the advanced veterinary equipment and care available in such hospitals^17^. The largest heatstroke study in dogs to date included 126 dogs presenting to a hospital in Israel and reported a case fatality rate of 53%^10^. That study used a retrospective case series analysis from a referral hospital population, preventing extrapolation of fatality rate to the wider canine population because of the inherent referral bias^17^. Consequently, results from such referral studies are not representative of the general canine population, reducing the generalisability and wider world application of the findings^18^.

In recent years, there has been considerable development of ‘Big Data’ databases combining primary-care clinical records from hundreds or even thousands of individual veterinary practices^19–21^. In the UK, VetCompass^TM^ has developed an online research platform that provides access to de-identified veterinary patient records from over 15 million companion animals and has been validated as a research resource by 75 peer reviewed publications to date^18,20^.

## Study aims

The current study aimed to use the VetCompass database of veterinary health records to (i) estimate the 2016 incidence of HRI in the UK dog population; (ii) identify canine risk factors for HRI and (iii) estimate case-fatality rate for HRI in dogs under primary veterinary care in 2016. It was hypothesised that brachycephalic breeds (specifically the Bulldog) have higher odds of HRI compared to mesocephalic breeds.

## Methods

### Data collection and management

The study used data from the VetCompass Programme that provides research access to de-identified electronic patient records (EPRs) from primary-care veterinary practices in the UK as previously described^22–25^. The study population included all dogs under primary veterinary care during 2016 in VetCompass. Dogs under veterinary care were defined as those with either a) at least one EPR recorded during 2016 and/or b) at least one EPR recorded during both 2015 and 2017. Data fields available for each dog included a unique animal identifier with breed, sex, neuter status, date of birth and bodyweight, and also clinical information from free-form text clinical notes, treatments and deceased status with relevant dates.

### Database search

#### Pilot study

Pilot investigations were conducted to refine the search terms used to identify candidate HRI cases within the denominator population. Because HRI is neither a definitive diagnosis nor a disorder that can be objectively confirmed through diagnostic testing, the case definition needed to be broad enough to include diagnoses reached by excluding other differential diagnoses and by consideration of the animal’s recent history. The final HRI case definition included any dog with strong evidence for HRI recorded in the EPR including a final stated diagnosis or insurance claim for a heat related illness (including terms such as heatstroke, heat stress, heat exhaustion or overheating) and/or a history of at least one of the following clinical signs developing specifically after, and being ascribed to, exposure to a hot environment, physical exertion or both.

Clinical signs:

- panting excessively or continuously despite removal from heat/cessation of exercise,
- collapse not subsequently attributed to another cause (e.g. heart failure, Addison’s),
- stiffness, lethargy or reluctance to move,
- gastrointestinal disturbance including hypersalivation, vomiting or diarrhoea,
- neurological dysfunction including ataxia, seizures, coma or death,
- haematological disturbances including petechiae or purpura.

Exclusion criteria included:

- subsequent diagnosis of an infectious or inflammatory condition that was not attributed to primary heat exposure such as kennel cough, pyometra or infectious meningitis,
- HRI or synonym listed only as one of a differential list,
- an earlier diagnosis of HRI that was later revised to exclude HRI, for example the dog was diagnosed with epilepsy following further seizure activity.

There are currently no explicit guidelines in dogs for accurately staging heat related illness and dogs, like humans, may progress through the stages of HRI depending on their treatment^26^. The HRI case definition in the current study included all stages of disease from mild (classically referred to as heat stress) to severe (classically referred to as heatstroke)^2^.

#### Main study

Candidate cases of HRI were identified by searching the EPR free-text fields for the following terms: heat stroke~3, heatst*, hyperthermi*, overheat*, over heated~2, heat exhaustion~2, hot car~2, collapse* + heat, cooling, high ambient temp*. Dogs identified from all searches were merged and randomly ordered. All candidate cases were manually reviewed in detail by two researchers (author 1 and author 2) to identify all confirmed HRI cases that met the study case definition for HRI occurring at any date within the patient’s available lifetime EPR (*prevalent HRI cases*), up to the point of data extraction (20^th^ January 2019). From these prevalent cases, the subset of *incident 2016 HRI cases* was identified with HRI events occurring only within the 2016 study period. All confirmed prevalent cases underwent further data extraction including outcome of event (survival or death) and date of heat exposure event. The first event occurring during the study period (2016) was used for the date of exposure event to calculate age at event for dogs with multiple HRI events.

### Analysis

Sample size calculations using Epi Info 7 were based on an estimated HRI incidence of 0.29% derived from a survey of the UK veterinary profession that reported an average of five canine HRI cases per practice during 2016^14^. There were approximately 5,000 small animal or mixed practices in the UK during 2016^27^, resulting in an estimated 25,000 HRI events within the 8.5 million dogs in the pet UK population^28^. The Bulldog has previously been reported at greater HRI risk than other dogs (odds ratio (OR) 2.7)^11^, and comprise 0.36% of the UK dog population^25^. Sample size calculations estimated that cross-sectional analysis would require 114,588 dogs (including 1879 Bulldogs) to provide a 2.7 odds ratio estimate for a disorder expected to occur in 0.29% of overall population with a 0.01% confidence limit and 90% power (60:1 ratio of control to exposed).

The study used a cohort design. The denominator population included 905,543 dogs from the VetCompass database. Demographic data were extracted automatically from the database for all study dogs and exported into Microsoft Excel (Office 365) for cleaning and descriptive analysis.

The prevalence of HRI within the cohort was estimated using all confirmed *prevalent HRI cases*. The one-year (2016) incidence was calculated using only *2016 incident HRI cases*. The event fatality rate was calculated using all dogs with at least one HRI event occurring in 2016. The 95% confidence intervals (CI) were calculated using EpiTools (AusVet 2019). The number of incident cases each month was plotted against the mean monthly UK air temperature, retrieved from the Met Office (https://www.metoffice.gov.uk/research/climate/maps-and-data/uk-and-regional-series) to provide a visual representation of the relationship between ambient temperature and HRI incidence.

Risk factor analysis used cohort clinical data to classify dogs as a case (*2016 incident HRI cases*) or non-case (all dogs in the denominator population not defined as a *2016 incident HRI case*) for HRI during the 2016 study period. Risk factor analysis was conducted in SPSS v25 using multivariable logistic regression to identify potential risk factors associated with HRI (defined in Table 1). Binary logistic regression was used to evaluate potential univariable associations between risk factors (*breed type, purebred, skull shape, adult bodyweight, bodyweight relative to breed/sex mean, sex/neuter* and *age*) and HRI diagnosis during 2016. As *breed type* was a factor of primary interest for the study, variables that are highly collinear with breed (*purebred*) or considered a defining characteristic of individual breeds (*adult bodyweight* and *skull shape*) were used in alternative models and not included in the multivariable models using *breed type,* as previously described^22^. Risk factors with liberal associations in univariable modelling (P < 0.2) were selected for multivariable evaluation. Model development used manual backwards stepwise elimination. Pairwise interactions were tested for all variables in the final multivariable model. The area under the receiver operating characteristic (ROC) curve was used to evaluate the predictive ability of the model^29^ alongside consideration of the underpinning biological plausibility of the model specification. Statistical significance was set at P < 0.05.

**Table 1:** Potential risk factors assessed for association with heat related illness (HRI) in UK dogs.

Potential risk factor for HRI	Variable definition	Justification
*Breed type*	Categorical variable including all named breed types (including both KC recognised purebred and non-KC recognised purebred) and designer hybrid types with contrived names (e.g. Cockapoo, Labradoodle, Lurcher) with ≥5 HRI cases and/or ≥5000 dogs in the overall study population. All remaining dogs were assigned to grouped categories of “other purebred”, “other designer cross” or “non-designer crossbred”.	.Belgian Malinois, Golden Retrievers and brachycephalic breeds are reported to have increased odds ratio of HRI compared to small breeds of dog^11^. Labrador Retriever was used as the comparator for this variable as they were the largest breed type in the denominator population (after crossbred) so enabled high statistical power to explore breed risks^29,30^
*Purebred*	Categorical variable grouping all dogs of recognisable breeds as “purebred”, all recognisable designer crossbreeds as “designer cross” and the remaining dogs as “crossbred”.	Purebred dogs are more likely to have an exaggerated conformation such as brachycephaly, thick coat, or giant body size, limiting their ability to thermoregulate^31^. A higher percentage of purebred dogs presented with heatstroke to one veterinary hospital^12^.
*Skull shape*	Purebred dogs were categorised by skull shape into three groups, “brachycephalic”, “mesocephalic” and dolichocephalic” (see Supplementary Note 1 for breeds by category). Designer crossbred dogs including a brachycephalic breed were classified as “brachycephalic cross” and all other dogs listed as crossbred or unrecorded breed were classified as “skull shape unrecorded”.	.Surface areas of the nasal turbinates and effective ventilation provide the mechanism to enable evaporative heat loss through panting, thus brachycephalic dogs have reduced heat dissipation mechanisms^11,31–33^
*Adult bodyweight*	Adult bodyweight was defined as the mean of all bodyweight (kg) values recorded for each dog after reaching 18 months old. Bodyweight (kg) was then categorised into seven groups (<10, 10-<20, 20-<30, 30-<40, 40-<50, ≥50), dogs under 18 months or with no recorded adult bodyweight were classified as “unrecorded”.	Small breeds of dog are reported to have decreased risk of HRI^11^, dogs with greater body mass have been reported to develop higher post exercise body temperatures^34^.
*Bodyweight relative to breed/sex mean*	A categorical variable grouping dogs with a mean adult bodyweight “equal or above” or “below” the mean adult bodyweight for their breed and sex (calculated using the overall VetCompass study population). An “unrecorded” variable included all dogs with no adult bodyweight or labelled as crossbred.	Increased bodyweight can be due to increases in either lean muscle mass, or body fat. Obesity limits heat conduction and radiation from the skin and can limit effective cooling via respiration^31^, overweight animals overheat faster and take longer to cool^35^. Dogs with greater lean body mass developed higher post exercise temperatures than lighter dogs^34^.
*Sex/neuter*	Dogs were classified by sex and neuter status into five categories (female entire, female neutered, male entire, male neutered) with “unrecorded” was used to group any dogs with no recorded sex or neuter status.	Male dogs develop higher body temperature post exercise^34,36^, and are over represented in cases of heatstroke presenting to veterinary hospitals^10,13,37^.
*Age*	The age variable described the age of the dog at the end of the study period (31^st^ December 2016) for non-case dogs, or the age at the first HRI event for *2016 incident HRI cases*. Age (years) was categorised into eight groups (<2, 2-<4, 4-<6, 6-<8, 8-<10, 10-<12, ≥12) with “unrecorded” for any dogs with no date of birth recorded in the EPR.	Older animals are more likely to have pre-existing conditions that limit effective heat dissipation such as heart disease, or respiratory diseases e.g. laryngeal paralysis^32^.

## Results

The study included 905,543 dogs under veterinary care at 886 UK VetCompass clinics during 2016. EPR searches identified 6531 candidate cases, of which 1222 were classified as *prevalent HRI cases* following manual review giving an estimated prevalence of 0.14% (95% CI 0.13-0.14%). There were 35 dogs identified with two HRI events and one dog with three recorded HRI events from the *prevalent HRI cases*. Data completeness varied between the variables assessed: *breed type* 99.55%, *sex/neuter* 99.53%, *age* 98.63% and *adult bodyweight* 65.70%.

### Incidence estimate

**Figure 1:**
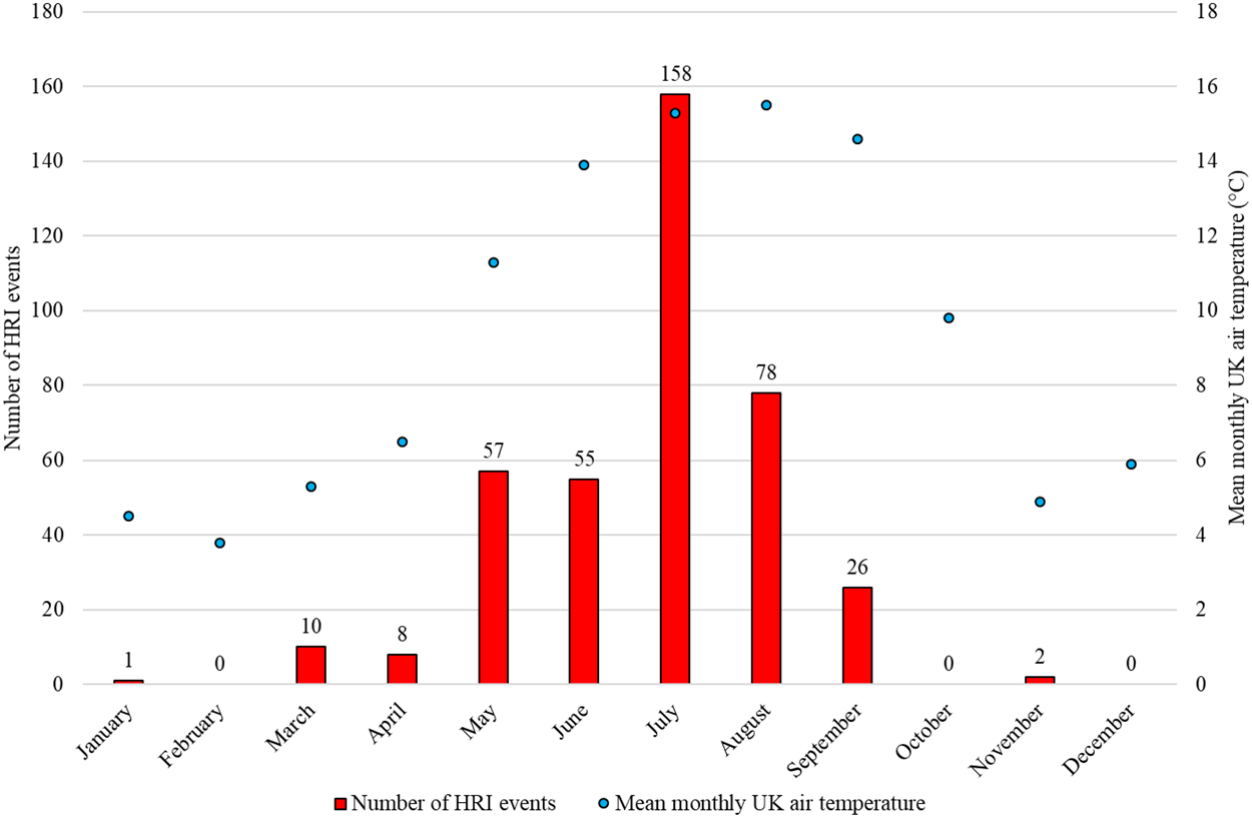
Heat-related illness cases by month for UK dogs under primary veterinary care at practices in the VetCompass Programme, against mean monthly UK air temperature for 2016.

There were 395 HRI events recorded in 2016 from 390 individual dogs. The incidence risk of HRI in dogs under primary veterinary care during 2016 was 0.04% (95% CI 0.04-0.05%). There were no HRI events during February, October or December, while 40% (158/395) of the incident cases were in July (see Fig. 1), corresponding with a heatwave event thought to have been triggered by a particularly strong El Nino^38^.

**Figure 2:**
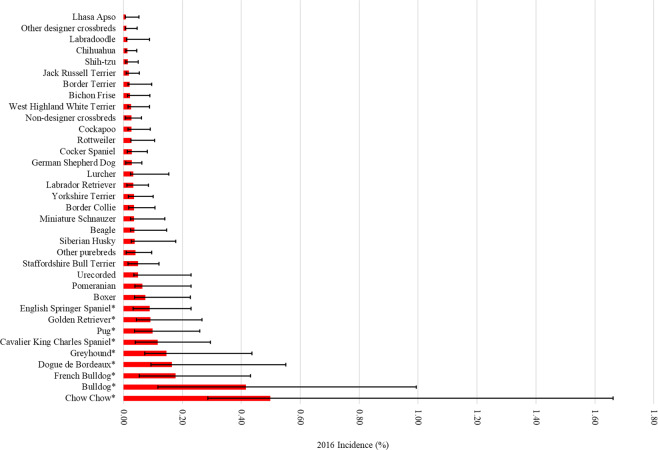
One-year (2016) incidence risk of heat related illness in dog breeds and designer crossbreeds under primary veterinary care at practices in the VetCompass Programme in the UK. The error bars show the 95% confidence interval. *Indicates breeds with increased odds identified by multivariable regression analysis.

**Figure 3:**
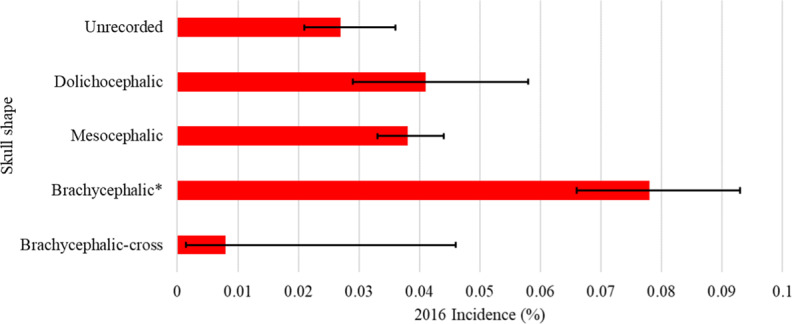
One-year (2016) incidence risk of heat related illness by Skull shape in dogs under primary veterinary care at practices in the VetCompass Programme in the UK. The error bars show the 95% confidence interval. *Indicates skull shapes with increased odds identified by multivariable regression analysis.

Breeds with the highest incidence of HRI were the Chow Chow (0.50%, 95% CI 0.21-1.16%), Bulldog (0.42%, 95% CI 0.30-0.58%, French Bulldog (0.18%, 95% CI 0.12-0.25%), Dogue de Bordeaux (0.17%, 95% CI 0.07-0.39%), Greyhound (0.15%, 95% CI 0.07-0.29%) and Cavalier King Charles Spaniel (0.12%, 95% CI 0.08-0.18%) (Fig. 2). The incidence risk of HRI in brachycephalic breeds overall was 0.08% (95% CI 0.07-0.09%) (Fig. 3).

### Fatality

During the 2016 period, 56 of the 395 HRI events resulted in death of the dog. The manner of death was not recorded for two cases. Of the remaining 54 deaths, 35 (64.81%) were by euthanasia and 19 (35.19%) were unassisted deaths. The event fatality rate for HRI in dogs during 2016 was 14.18% (95% CI 11.08 – 17.96%).

### Risk analysis

Univariable binary logistic regression modelling identified *breed type* (R^2^ = 0.040, P < 0.001), *purebred* (R^2^ = 0.004, P < 0.001)*, skull shape* (R^2^ = 0.009, P < 0.001), *adult bodyweight* (R^2^ = 0.006, P < 0.001), *bodyweight relative to breed/sex mean* (R^2^ = 0.002, P < 0.001) and *age* (R^2^ = 0.002, P = 0.063) as factors liberally associated with HRI, but not *sex/neuter* (R^2^ = 0.001, P = 0.459) (see Supplementary Note 2 for descriptive and univariable regression results).

The final breed multivariable model retained three risk factors: *breed type, bodyweight relative to breed/sex mean* and *age* (R^2^ = 0.045, degrees of freedom = 43). The model showed acceptable discrimination (area under the ROC curve: 0.718). In the final model (Table 2), nine breeds (Chow Chow, Bulldog, French Bulldog, Dogue de Bordeaux, Greyhound, Cavalier King Charles Spaniel, Pug, English Springer Spaniel and Golden Retriever) had higher odds of HRI compared to Labrador Retrievers. Crossbreds were not at significantly different odds compared to Labrador Retrievers (OR 0.82, 95% CI 0.49-1.37, p = 0.450). No breed types had significantly reduced odds of HRI compared to Labrador Retrievers. Dogs with bodyweight equal to or greater than the relative breed/sex mean had higher odds of HRI (OR 1.42, 95% CI 1.12-1.80) compared to dogs weighing below the relative breed/sex mean. Dogs in the 2-<4 years, 6-<8 years and ≥12 years categories had greater odds compared to dogs <2 years old, dogs ≥12 years had the greatest odds of HRI (OR 1.75, 95% CI 1.14-2.70).

**Table 2:** Multivariable binary logistic regression results for risk factors associated with heat related illness in dogs under primary veterinary care in the VetCompass Programme in the UK during 2016.

Independent Variable	Odds Ratio	95% CI	P-value
*Breed type*			<0.001
Labrador Retriever	Base		
Chow Chow	16.61	6.21–44.44	<0.001
Bulldog	13.95	8.01–24.29	<0.001
French Bulldog	6.49	3.62–11.63	<0.001
Dogue de Bordeaux	5.31	1.99–14.21	0.001
Greyhound	4.26	1.88–9.70	0.001
Cavalier King Charles Spaniel	3.45	1.86–6.42	<0.001
Pug	3.24	1.67–6.29	<0.001
English Springer Spaniel	2.74	1.25–6.01	0.012
Golden Retriever	2.65	1.40–5.01	0.003
Boxer	2.26	0.95–5.34	0.064
Pomeranian	2.12	0.48–9.30	0.319
Missing	2.11	0.72–6.20	0.173
Staffordshire Bull Terrier	1.50	0.84–2.68	0.175
Other purebred	1.28	0.76–2.14	0.358
Siberian Husky	1.18	0.28–5.06	0.822
Beagle	1.15	0.34–3.86	0.827
Miniature Schnauzer	1.08	0.32–3.64	0.900
Border Collie	1.08	0.47–2.44	0.862
Yorkshire Terrier	1.05	0.49–2.25	0.898
Lurcher	1.00	0.24–4.30	0.996
German Shepherd Dog	0.92	0.35–2.48	0.874
Cocker Spaniel	0.88	0.35–2.20	0.788
Rottweiler	0.86	0.20–3.69	0.841
Cockapoo	0.85	0.39–1.87	0.686
Non-designer crossbred	0.82	0.49–1.37	0.450
West Highland White Terrier	0.76	0.28–2.02	0.581
Bichon Frise	0.68	0.20–2.27	0.525
Border Terrier	0.61	0.14–2.60	0.503
Jack Russell Terrier	0.55	0.25–1.21	0.135
Shih-tzu	0.47	0.18–1.24	0.127
Chihuahua	0.44	0.17–1.18	0.102
Labradoodle	0.41	0.06–3.06	0.384
Other designer crossbred	0.33	0.08–1.41	0.133
Lhasa Apso	0.24	0.03–1.76	0.159
*Age*			0.085
<2 years	Base		
2-<4 years	1.56	1.13–2.14	0.007
4-<6 years	1.41	0.99–2.02	0.058
6-<8 years	1.53	1.05–2.23	0.026
8-<10 years	1.21	0.79–1.86	0.374
10-<12 years	1.16	0.71–1.88	0.559
≥12 years	1.75	1.14–2.70	0.011
Unrecorded	0.70	0.22–2.26	0.552
*Bodyweight relative to breed/sex mean*			0.001
Below	Base		
At or above	1.42	1.12–1.80	0.004
Unrecorded	0.91	0.69–1.20	0.505

As described in the methods, variables collinear (*purebred*) and definitive of breed types (*bodyweight* and *skull shape*) replaced the *breed type* variable in the final multivariable model (Table 3). Purebred dogs had 1.86 times (95% CI 1.39-2.49) the odds of crossbred dogs. Brachycephalic dogs had higher odds of HRI (OR 2.10, 95% CI 1.68-2.64) compared to mesocephalic dogs. Dogs over 50 kg in bodyweight had 3.42 times the odds of HRI (95% CI 1.54-7.57) compared to dogs weighing under 10 kg.

**Table 3:** Results for variables that individually replaced the *breed type* variable in the final multivariable logistic regression model (with *age* and *bodyweight relative to breed/sex mean*) to evaluate risk factors associated with heat related illness in dogs under primary veterinary care in the VetCompass Programme in the UK during 2016.

Variable	Odds ratio	95% CI	P-value
*Skull shape*			<0.001
Mesocephalic	Base		
Dolichocephalic	1.08	0.74–1.58	0.698
Brachycephalic-cross	0.22	0.03–1.56	0.129
Brachycephalic	2.10	1.68–2.64	<0.001
Unrecorded	0.73	0.53–0.99	0.039
*Purebred*			<0.001
Crossbred	Base		
Designer Cross	0.72	0.36–1.41	0.337
Purebred	1.86	1.39–2.49	<0.001
Unrecorded	2.41	0.57–10.16	0.233
*Bodyweight (kg)*			<0.001
<10	Base		
10-<20	1.82	1.31–2.53	<0.001
20-<30	2.13	1.51–3.02	<0.001
30-<40	1.74	1.14–2.65	0.010
40-<50	1.76	0.91–3.40	0.093
≥50	3.42	1.54–7.57	0.002
Unrecorded	0.65	0.16–2.70	0.553

## Discussion

This is the largest primary-care study to report the incidence, risk factors and case fatality for HRI in dogs in the UK. The 0.04% annual incidence of HRI in dogs under primary veterinary care based on our real clinical records is considerably lower than the incidence predicted by an opinion survey of veterinary surgeons carried out during the same study period (2016)^14^. This highlights the issue of recall bias when using surveys based on belief rather than documented reports of disorders, especially following exposure to media campaigns highlighting hazards that may promote a recency effect to encourage higher ‘recall’^39^. That survey was conducted following the launch of the “Dogs die in hot cars” campaign^40^, which could explain the ‘prompted’ high numbers of cases reported by veterinary surgeons. It should also be noted that the survey included all veterinary surgeons, including those working in referral and out-of-hours emergency hospitals, which may also contribute to the high numbers of cases reported, whereby individual cases could be double, or triple counted.

The current study reports event fatality for HRI events at 14.18%. Our primary-care case fatality rate is lower than previous reports of 36–50%^11–13^. However, ours is the first study to include all stages of HRI presentation, including early stages which may be managed without hospitalisation, while previous studies tended to include only the more severely affected subset of dogs defined as having “heatstroke” or advanced heat related illness presenting to referral hospitals. This is the first study to use a primary veterinary care population rather than a referral population, highlighting the limitations of using referral populations to estimate incidence and fatality at a population level^17^. The relatively low fatality rate reported in the current study could also reflect the temperate UK climate. As previous studies have primarily been conducted in countries with hotter climates such as Israel^11–13^, the comparatively higher fatality rates reported in hotter countries should serve as a warning for the UK. As climate change accelerates both the frequency and severity of heat waves^8^, the incidence and fatality rate of HRI is likely to increase in dogs^7^. In the current study, 40% of the incident cases were in July, corresponding to a European heat-wave. This 2016 July heat-wave was attributed to the El Nino effect and was previously the hottest July on record^38^. This record has since been broken; July 2019 is currently the hottest on record without the contribution of a particularly strong El Nino effect, highlighting the impact global warming is having on extreme temperature events^38^. The number of days per year with extreme heat in Europe have tripled in the last two decades, prompting concerns that global warming is accelerating even more rapidly than predicted^41^.

Compared to Labrador Retrievers in the final multivariable model, nine breeds had significantly higher odds of HRI: Chow Chow, Bulldog, French Bulldog, Dogue de Bordeaux, Greyhound, Cavalier King Charles Spaniel, Pug, English Springer Spaniel and Golden Retriever. These findings agree with previous studies that reported increased HRI risk to brachycephalic breed types in general^10^, Bulldogs^11,12^ and Golden Retrievers^11^. The increased odds ratios for these breeds in the multivariable compared to the univariable models highlights the importance of undertaking multivariable analysis when investigating breed related risk studies^29^. As well as some extremely brachycephalic breeds (Bulldog and French Bulldog), we also identified increased risk in some mesocephalic breeds. The Golden Retriever had 2.67 times the odds compared to the Labrador Retriever despite being of similar size, temperament and purpose. The Golden Retriever does, however, have a thicker coat than the Labrador which may be the tipping factor for HRI between these two breeds. The Chow Chow, another breed with a very thick coat, had the greatest risk of HRI of all the study breeds (OR = 16.17), although it must be noted the confidence interval for this breed is very wide due to the relatively small sample size. Coat type is potentially an important risk factor for HRI in dogs, but may instead be a complicating factor for dogs with a behavioural or anatomical (e.g. brachycephaly) predisposition to overheating. Other double coated breeds may also have increased risk, however the current study was under-powered to fully explore these pre-dispositions due to low numbers of these breeds.

The Labrador Retriever has previously been reported with increased risk of HRI^11^, although that study included both Labrador and Golden Retrievers as a single breed and therefore the increased risk may have come from the subset of these dogs that were Golden Retrievers^11^. The current study elected to use the Labrador Retriever as the comparator breed instead of crossbred for similar reasons to those suggested by Erlen *et al*. (2018)^30^. Crossbred dogs show a high level of variability in genetics, bodyweight, skull shape and conformation compared to a single specified purebred breed^42^. This high variability challenges the utility of crossbreds as a standard comparator breed. Second only to crossbreds, the Labrador Retriever was the most common breed in the current study, comprising 6.6% of the total study population and enabling high statistical power to explore breed related risk^29^. In the current study, the odds of HRI were not significantly different between Labrador Retrievers and crossbred dogs.

It has been suggested that racing Greyhounds rarely exhibit HRI^43^ but our results showed pet Greyhounds to have 4.26 times the odds of HRI when compared to the Labrador Retriever. As a dolichocephalic breed, they are not commonly associated with obesity^44^, however they tend to have a high lean muscle mass which has been associated with increased risk of post exercise hyperthermia^34^. Further research is required to explore the underlying reason for this result, but a recent study found “collapse” to be the second most common cause of death in Greyhounds, specifically in animals over 12 years old^44^. A degenerative cardiovascular or respiratory disorder (such as laryngeal paralysis) could potentially promote their increased risk of HRI.

This study included three grouped breed-type variables (other purebred, other designer crossbred and crossbred) and 32 individual breeds and designer hybrid breed types, because breed was a factor of primary interest. Including this many breed-type variables increased the degrees of freedom in the regression model, negatively impacting the statistical power. However, it is important to include as many popular breeds as possible in risk analysis studies to enable identification of breed predispositions to disorders, but also to identify breeds with apparent resistance or protection from disorders. HRI is a disorder that requires extrinsic input; dogs do not develop HRI due an underlying intrinsic factor (unlike osteoarthritis or cancer) and non-disease is the natural internal state. To develop HRI dogs, must be exposed to either a hot environment and/or an activity that induces significant hyperthermia. In support of this theory, there were no individual breed types identified with significantly reduced risk of HRI compared to the Labrador Retriever in this study. Additionally, whilst the multiple regression model including breed-type showed acceptable discrimination, the model R^2^ value (0.045) is very low, suggesting there are key (likely non-canine) factors missing from this model such as ambient temperature and the dog’s acclimatisation to heat. The canine risk factor model explored in this study should be considered explanatory, identifying canine features than increase a dog’s risk of HRI, rather than predictive. A predictive HRI model would need to include additional non-canine variables such as ambient temperature, humidity, activity levels, fitness and hydration^36^.

This study supports assertions that brachycephaly is associated with increased risk of HRI in dogs. Five of the nine breeds with significantly increased odds of HRI were brachycephalic (Bulldog, French Bulldog, Dogue de Bordeaux, Cavalier King Charles Spaniel and Pug), additionally the Boxer (OR 2.26, 95%CI 0.95-5.34), another brachycephalic breed trended towards increased odds. Compared to mesocephalic dogs in multivariable modelling, brachycephalic dogs had 2.10 times the odds of HRI, with no significant difference in HRI odds between dolichocephalic and mesocephalic dogs. Interestingly, brachycephalic crosses had the lowest incidence of HRI across all skull types including unrecorded (including predominantly crossbred dogs) (see Fig. 2). They did not however, have a significantly reduced odds ratio of HRI at multivariable level. This could potentially be due to this group of dogs being comparatively young compared to the rest of the population^45^, but could also be attributed to the typically lower bodyweight of designer crossbreds. The mean designer cross bodyweight in this study was 15.6 kg, compared to the mean purebred bodyweight of 17.5 kg, and mean crossbred bodyweight of 17.4 kg. This finding could lend support to the argument for outbreeding to increase muzzle length within extreme brachycephalic breeds to improve the health and welfare of dogs^46^ but requires further research.

Dogs at or above the mean adult bodyweight for their breed/sex showed increased risk of HRI compared to dogs below the mean bodyweight in multivariable analysis. Increased HRI risk in heavier individuals within breeds agrees with findings from previous studies exploring post-race body temperature in Greyhounds; dogs with a greater lean body mass were significantly hotter post-race than lighter dogs^34^. It was not possible to determine whether elevated bodyweight within breed was due to obesity, conformation differences or increased muscle mass in this study as body condition scores were not available from the EPRs. There are several different scales used for measuring body condition score in dogs, meaning it is not possible to make direct comparisons between clinical records when a single unit is used to record a dog’s score. As highlighted in a report by Ward et al.^47^, adopting an single universal score for body condition score in dogs should be considered a key priority for veterinary professionals and canine welfare organisations to allow consistent measurement, recording and monitoring of body composition in dogs.

Dogs weighing over 50 kg in absolute bodyweight had the highest odds of HRI, and all bodyweight groups over 10 kg had significantly higher odds for HRI than dogs weighing under 10 kg. Smaller dogs have a high heat storage to radiative surface area ratio that results in more rapid heat loss compared to larger dogs, meaning they can exercise for longer before overheating^48,49^. This increased efficiency in radiative heat loss could explain why the purebred breeds with the lowest odds of HRI are all small breed dogs (Table 2). Of the breeds analysed, the Lhaso Apso, Shih tzu and Chihuhua had the lowest odds of HRI despite all being brachycephalic breeds. These breeds all typically weigh under 10 kg. In comparison, French bulldogs and Cavalier King Charles Spaniels typically weigh over 10 kg^45,50^, whilst Pugs typically weigh around 10 kg;^51^ the HRI odds for these three breeds appears to be proportional to their typical bodyweights.

Reflecting the findings of Drobatz and Macintire (1996), purebred dogs showed increased risk of HRI compared to crossbred dogs in the current study. Purebred dogs are more likely to have exaggerated features such as thick coats, extreme body size and skull shapes^31^, all of which were predicted, and subsequently appear to, impact HRI risk. In comparison, wild dogs such as the African Hunting dog, have been shown to tolerate higher core body temperatures than domestic dogs during exercise in hot climates (41 °C), and lose a greater proportion of the heat generated through non-evaporative mechanisms effectively conserving water^52^.

In the final multivariable model, dogs ≥12 years old had the greatest odds of HRI compared to dogs <2 years old, reflecting the human literature which reports an increased HRI risk to humans of advanced age or with chronic medical conditions^53^. Dogs with pre-existing medical conditions were excluded from previous heatstroke studies^11,12,54^. Older dogs are more likely to be affected by age related conditions such as respiratory or cardiovascular disease, therefore elderly dogs were potentially absent from the previous study populations and subsequent analysis. Old age in humans is associated with increased HRI risk, due to a decreased physiological ability to dissipate heat (notably changes in sweat production and decreased skin blood flow)^55^. Further studies should explore the effect of age on canine thermoregulatory ability.

Despite previous studies suggesting male dogs have increased risk of HRI^34,36^, there was no significant difference in odds for HRI between males and females, or between neutered and entire dogs shown in the current study. Previous HRI studies have reported mixed findings for sex as a risk factor in dogs, varying from no reported difference^11,16^ to male overrepresentation^10,13,37^. As this is the first study to include multivariable analysis for HRI risk factors in dogs, it is possible that including relative bodyweight in the analysis accounted for the possible confounding effect of increased bodyweight in males.

Alongside the environmental threat to canine health and welfare, the canine population itself is becoming less heat tolerant as proportional obesity^56^ and brachycephalism increase^25,45,51^. The current study found that brachycephalic breeds comprised 18.42% of the total primary-care population in 2016 and the KC reported that the French Bulldog became the most commonly registered pedigree breed in 2018^57^. Increasing popularity of brachycephalic breeds coupled with the increasing frequency of heatwave events poses a significant welfare concern for the modern dog population and is likely to result in increasing incidence of HRI over the coming years.

A recent survey of brachycephalic dog owners found over a third reported that their dog had a problem with heat regulation^58^. The survey did not differentiate simple overheating from overheating leading to HRI, but nonetheless identified heat regulation as the most common problem perceived by brachycephalic owners^58^. The comparably lower incidence of HRI reported in this study compared to the owner reports of overheating in the survey conducted by Packer *et al*.^58^ could be due to several possibilities. Owners of brachycephalic dogs may normalise heat related issues in these breeds and therefore not perceive overheating to be of sufficient concern to either report events or seek treatment from their veterinary surgeon. Similarly, veterinary surgeons may normalise these findings such that they do not consider these events significant enough to warrant recording in the dog’s clinical notes. Alternatively, owners of brachycephalic dogs may be more aware of their dog’s risk of overheating and take deliberate actions to prevent occurrence, thus preventing their dogs developing severe HRI. Overall, it is probable that the true incidence of HRI in UK dogs, particularly brachycephalics, is likely to be higher than estimated in this current study.

There were some limitations to the study. The study used manual stepwise elimination to select the final breed model, despite mounting opposition to this method of model selection^59–61^. Whilst the automated stepwise method fails to take into account prior knowledge and risks artificially elevating R^2^ values, this study included consideration of biological plausibility when selecting the final model variables and aimed to produce an explanatory model, rather than a predictive model.

As noted in a previous study, the data used in this study were not recorded for research purposes meaning there are likely to be missing data within the dataset^24,62^. Additionally, the inclusion criteria for an HRI case relied upon the accuracy and completeness of the clinical notes associated with the event. As dogs with HRI typically present as an emergency, clinical notes may be less accurately recorded at the time of treatment meaning false negatives are more likely^63^. The lack of a definitive diagnostic test for HRI meant that by necessity, the inclusion and exclusion criteria for an HRI case could have resulted in some dogs being mis-identified. Thus, for cases where owner treatment preference or finances prevented further diagnostic testing to rule out other non-HRI causes of hyperthermia, these cases could have been misidentified as HRI cases. The study population comprised only dogs under the care of primary veterinary practices, many of which do not offer a full 24-hour emergency service. Whilst it is likely that some cases presented to dedicated out of hours emergency practices which were not included in this study, most out of hours practices send clinical notes on to the patient’s first opinion practice so most would still have been included in this study. Additionally, HRI often follows failure of the dog owner to appreciate the risk of a hot environment or of undertaking extreme exercise to the dog, meaning there could often be elements of owner guilt associated with the condition. This may reduce the willingness of an owner to declare the true reason for the dog’s illness or death. As previously noted, overheating may be perceived by owners of some breeds as normal or may be managed by the owner, meaning HRI events are not reported or presented to the dog’s primary care practice. These limitations combined with the lack of a definitive diagnostic test or definition for HRI suggest that the true incidence of HRI may be much higher than reported in this study.

The current study defined specific breeds by skull morphology as described in the methods and Supplementary Material. Although the breeds assigned to each skull type category could reasonably be challenged, changing the specific breeds listed in each category is unlikely to change the overall inferences drawn from the analysis^64^. The traditional definitions used to describe canine skull shape with three categories from dolichocephalic to brachycephalic is potentially oversimplified, and the newer cephalic index systems derived from various skull width to length ratios may be more accurate^65^. However the variability in cephalic index between individuals of the same breed and between sexes^65,66^, and the limited widescale measurement of cephalic index in large numbers of different breeds means it was not possible to use this measurement in place of the traditional skull morphology definitions for the current study. Whilst coat type has been noted as another possible risk factor for HRI in dogs, it was not possible to accurately retrieve coat type for individual dogs from the EPRs in this study, as this is not a characteristic commonly recorded by veterinary practice management software and can often vary widely within individual breeds.

Finally, the current study population included only dogs under care of a primary veterinary practice during the study period of 2016. It was therefore not possible to include HRI events prior to, or after 2016 in the risk factor analysis as this would introduce selection bias because fatal HRI events prior to 2016 would be excluded, and dogs born after 2016 would be excluded from events post 2016. This limited the number of HRI cases for analysis and prevented any investigation into changes in HRI incidence over time. These could be considered as research topics for a future study.

## Conclusions

Extreme heat events are predicted to increase in both frequency and severity, with deaths due to HRI in humans predicted to triple by 2050^7^. This is the first study to explore HRI in a large population of dogs under primary veterinary care and the canine risk factors identified suggest that similar risk factors for HRI apply for both humans and dogs. This study found that increased bodyweight (relative to breed), brachycephaly and age significantly increased the risk of HRI and identified nine breeds at significantly increased risk. Maintaining healthy bodyweight should be considered an important management tool for limiting HRI risk, therefore routine recording of patient body condition score should be highlighted as a key strategy for enabling the monitoring and subsequent management of canine obesity. Prevention is an incredibly important strategy for limiting the welfare implications and mortality caused by HRI in both man and dog. The results of this study can assist veterinary practitioners, breeders and owners in identifying dogs and breeds at greater risk of HRI and therefore with implementation of strategies to reduce the risks of HRI in dogs.

### Ethics approval

Ethics approval was granted by the RVC Ethics and Welfare Committee (reference number SR2018-1652).

## Supplementary information

Supplementary information.

## Data Availability

The datasets supporting the current study are publicly available on the RVC data repository: http://researchonline.rvc.ac.uk/id/eprint/12379/.
