# Supplementary material for: Incidence and risk factors for heat-related illness (heatstroke) in UK dogs under primary veterinary care in 2016
Source: Sci Rep. 2020 Jun 18;10:9128. doi: 10.1038/s41598-020-66015-8 (PMC7303136; doi:10.1038/s41598-020-66015-8)
Supplement: Supplementary file 1 — Supplementary information. [file 41598_2020_66015_MOESM1_ESM.docx]

**Incidence and risk factors for heat-related illness (heatstroke) in UK dogs under primary veterinary care in 2016.**

Authors:

Emily J. Hall*, MA VetMB PGCAP MRSB MRCVS FHEA, School of Animal, Rural and Environmental Science, Nottingham Trent University, Brackenhurst, Southwell, Notts NG25 0QF, UK.

Anne J. Carter, BSc (Hons) MSc PhD FHEA MRSB, School of Animal, Rural and Environmental Science, Nottingham Trent University, Brackenhurst, Southwell, Notts NG25 0QF, UK.

Dan G. O’Neill, MVB BSc(hons) MSc(VetEpi) PhD FRCVS, Pathobiology and Population Science, The Royal Veterinary College, Hawkshead Lane, North Mymms, Hatfield, Herts AL9 7TA, UK [doneill@rvc.ac.uk](mailto:doneill@rvc.ac.uk)

*Corresponding author: emily.hall@ntu.ac.uk

**Supplementary Note 1**: Breed types by skull shape.

Table 1. Breed types defined as brachycephalic skull shape.

| \| Brachycephalic breed types \| \| \| \| --- \| --- \| --- \| \| Affenpinscher \| Cavalier King Charles Spaniel \| Miniature Bulldog \| \| Alapaha Blue Blood Bulldog \| Chihuahua \| Miniture Shih-Tzu \| \| American Bandogge Mastiff \| Dogue de Bordeaux \| Neopolitan Mastiff \| \| American Bulldog \| Dorset Olde Tyme Bulldogge \| Old English Mastiff \| \| American Lo-Sze Pugg \| English Mastiff \| Olde Boston Bulldogge \| \| American Pocket Bully \| French Bulldog \| Olde English Bulldogge \| \| Australian Bulldog \| German Boxer \| Olde Victorian Bulldogge \| \| Aylestone Bulldog \| Griffon \| Pekingese \| \| Boston Terrier \| Griffon Bruxellois \| Pug \| \| Boxer \| Italian Mastiff \| Shih-tzu \| \| Bull Boxer \| Japanese Chin \| Spanish Bulldog \| \| Bull Mastiff \| King Charles Spaniel \| Valley Bulldog \| \| Bulldog \| Lhasa Apso \| Victorian Bulldog \| \| Catahoula Bulldog \| Mastiff \|  \| |  |  |  |
| --- | --- | --- | --- | --- | --- | --- | --- | --- | --- | --- | --- | --- | --- | --- | --- | --- | --- | --- | --- | --- | --- | --- | --- | --- | --- | --- | --- | --- | --- | --- | --- | --- | --- | --- | --- | --- | --- | --- | --- | --- | --- | --- | --- | --- | --- | --- | --- | --- |

Table 2. Breed types defined as brachycephalic designer-cross skull shape

| Brachycephalic-cross breed types | | |
| --- | --- | --- |
| American Lamalese | Chiweenie | Pugalier |
| Beaglier | Chug | Puganese |
| Boxador | Cockalier | Pugapoo |
| Boxoodle | Jackachi | Puggle |
| Bug | Jackashit | Pugland |
| Bullador | Jug | Pugpom |
| Cavachi | La-Chon | PugTzu |
| Cavachon | Lha-Cocker | Sharanian |
| Cavadoodle | Lhasalier | Sharpador |
| Cavador | Lhasapoo | ShiChi |
| Cavajack | Malshi | Shichon |
| Cavapom | Maltalier | Shih-Poo |
| Cavapoo | Pekepoo | Shiranian |
| Cavapoochon | Pomapug | Shorkie |
| Cavatzu | Pomchi | Shug |
| Chichon | Pooshit |  |
| Chi-Poo | Pugador |  |

Table 3. Breed types defined as dolichocephalic skull shape.

| Dolichocephalic breed types | | |
| --- | --- | --- |
| Afghan Hound | Dachsbracke | Miniature Pinscher |
| Airedale Terrier | Dachshund | Miniature Poodle |
| American Bull Terrier | Dashapoo | Peruvian Hairless |
| American White Shepherd Dog | Dutch Sheepdog | Petit Basset Griffon Vendeen |
| Andalusian Mouse-Hunting Dog | Dutch Shepherd Dog | Pharaoh Hound |
| Anglos-Francaises de Petite Venerie | English Bull Terrier | Pinscher |
| Ariege Pointer | English Greyhound | Podenco |
| Australian Kelpie | English Terrier | Podenco Canario |
| Azawakh Hound | Fox Terrier | Poodle |
| Basset Griffon Vendeen | French Basset | Portuguese Podengo |
| Basset Hound | German Guard Dog | Saarloos Wolfhound |
| Bavarian Mountain Hound | German Pinscher | Saluki |
| Beauce Shepherd Dog | German Shepherd Dog | Scottish Collie |
| Bedlington Terrier | German Shepherd Dog - White | Scottish Deerhound |
| Belgian Groenendael Shepherd Dog | Grand Basset Griffon Vendeen | Scottish Rough Collie |
| Belgian Laekenois Shepherd Dog | Grand Griffon Bleu de Gascogne | Scottish Smooth Collie |
| Belgian Malinois Shepherd Dog | Grand Griffon Vendeen | Scottish Terrier |
| Belgian Shepherd | Great Dane | Shetland Sheepdog |
| Belgian Short-Haired Pointer | Greyhound | Sloughi |
| Belgian Tervuren Shepherd Dog | Ibizan Hound | Smooth Fox Terrier |
| Black and Tan Coonhound | Irish Terrier | Spanish Greyhound |
| Bleu de Gascogne Basset | Irish Wolfhound | Spanish Hound |
| Bloodhound | Italian Greyhound | Spanish Podenco |
| Bluetick Coonhound | Italian Hound | Standard Doberman Pinscher |
| Borzoi | Italian Shepherd Dog | Standard Poodle |
| Bracco Italiano | Kerry Blue Terrier | Swiss White Shepherd Dog |
| Braque Francais | King Shepherd Dog | Teacup Poodle |
| British Wolfdog | Lakeland Terrier | Teckel Dachshund |
| Bruno Jura Hound | Lurcher | Toy Fox Terrier |
| Bull Terrier | Manchester Terrier | Toy Manchester Terrier |
| Carlin Pinscher | Mexican Hairless - Intermediate | Toy Poodle |
| Carpathian Sheepdog | Miniature Bull Terrier | Trail Hound |
| Chippiparai | Miniature Coloured Bull Terrier | Welsh Terrier |
| Cirneco Dell 'Etna | Miniature Dachshund | Whippet |
| Coonhound | Miniature Doberman Pinscher | Wire-Haired Fox Terrier |
| Croatian Sheepdog | Miniature English Bull Terrier | Wolfhound |
| Czesky Terrier | Miniature Fox Terrier | Yugoslavian Shepherd Dog |

All other named breed-types were classified as mesocephalic.

All crossbred, designer crossbred or dogs with no listed breed were classified as *unknown* skull shape.

**Supplementary note 2:** Univariable and descriptive statistics for potential risk factors.

Table 4. Descriptive and univariable logistic regression results for breed type associated with heat related illness in dogs under primary veterinary care in the VetCompass^TM^ Programme in the UK during 2016.

| Independent variable | Case No. (%) | Non-case No. (%) | Odds ratio | 95% CI | P-value |
| --- | --- | --- | --- | --- | --- |
| *Breed type* |  |  |  |  | <0.001 |
| Labrador Retriever | 20 (5.13) | 59,943 (6.62) | Base |  |  |
| Chow Chow | 5 (1.28) | 997 (0.11) | 15.03 | 5.63-40.13 | <0.001 |
| Bulldog | 35 (8.97) | 8375 (0.93) | 12.53 | 7.23-21.71 | <0.001 |
| French Bulldog | 29 (7.44) | 16,368 (1.81) | 5.31 | 3.00-9.39 | <0.001 |
| Dogue de Bordeaux | 5 (1.28) | 3027 (0.33) | 4.95 | 1.86-13.20 | 0.001 |
| Greyhound | 8 (2.05) | 5448 (0.60) | 4.40 | 1.94-10.00 | <0.001 |
| Cavalier King Charles Spaniel | 20 (5.13) | 17,237 (1.90) | 3.48 | 1.87-6.47 | <0.001 |
| Pug | 16 (4.10) | 16,198 (1.79) | 2.96 | 1.53-5.71 | 0.001 |
| English Springer Spaniel | 18 (4.62) | 20,190 (2.23) | 2.76 | 1.26-6.06 | 0.012 |
| Golden Retriever | 9 (2.31) | 9784 (1.08) | 2.67 | 1.41-5.05 | 0.002 |
| Boxer | 7 (1.79) | 9435 (1.04) | 2.22 | 0.94-5.26 | 0.069 |
| Pomeranian | 4 (1.03) | 6217 (0.69) | 1.93 | 0.66-5.64 | 0.231 |
| Missing | 2 (0.51) | 4054 (0.45) | 1.48 | 0.35-6.33 | 0.598 |
| Staffordshire Bull Terrier | 26 (6.67) | 53,029 (5.86) | 1.47 | 0.82-2.63 | 0.196 |
| Other purebred | 51 (13.08) | 123,018 (13.59) | 1.24 | 0.74-2.08 | 0.411 |
| Siberian Husky | 2 (0.51) | 5239 (0.58) | 1.14 | 0.27-4.90 | 0.856 |
| Beagle | 3 (0.77) | 8067 (0.89) | 1.12 | 0.33-3.75 | 0.861 |
| Miniature Schnauzer | 3 (0.77) | 8393 (0.93) | 1.07 | 0.32-3.61 | 0.911 |
| Border Collie | 8 (2.05) | 22,396 (2.47) | 1.07 | 0.47-2.43 | 0.870 |
| Yorkshire Terrier | 10 (2.56) | 28,169 (3.11) | 1.06 | 0.50-2.27 | 0.873 |
| Lurcher | 2 (0.51) | 6020 (0.67) | 1.00 | 0.23-4.26 | 0.995 |
| German Shepherd Dog | 6 (1.54) | 21,334 (2.36) | 0.84 | 0.34-2.10 | 0.714 |
| Cocker Spaniel | 9 (2.31) | 32,136 (3.55) | 0.84 | 0.38-1.84 | 0.663 |
| Rottweiler | 2 (0.51) | 7283 (0.80) | 0.82 | 0.19-3.52 | 0.793 |
| Cockapoo | 5 (1.28) | 18,247 (2.02) | 0.82 | 0.31-2.19 | 0.694 |
| Non-designer Crossbred | 52 (13.33) | 193,878 (21.42) | 0.80 | 0.48-1.35 | 0.407 |
| West Highland White Terrier | 5 (1.28) | 18,873 (2.09) | 0.79 | 0.30-2.12 | 0.645 |
| Bichon Frise | 3 (0.77) | 13,265 (1.47) | 0.68 | 0.20-2.28 | 0.530 |
| Border Terrier | 2 (0.51) | 9649 (1.07) | 0.62 | 0.15-2.66 | 0.521 |
| Jack Russell Terrier | 9 (2.31) | 48,426 (5.35) | 0.56 | 0.25-1.22 | 0.145 |
| Shih-tzu | 5 (1.28) | 32,905 (3.64) | 0.46 | 0.17-1.21 | 0.116 |
| Chihuahua | 5 (1.28) | 37,253 (4.12) | 0.40 | 0.15-1.07 | 0.069 |
| Labradoodle | 1 (0.26) | 7484 (0.83) | 0.40 | 0.05-2.98 | 0.372 |
| Other designer crossbred | 2 (0.51) | 20,268 (2.24) | 0.30 | 0.07-1.27 | 0.100 |
| Lhasa Apso | 1 (0.26) | 12,548 (1.39) | 0.24 | 0.03-1.78 | 0.162 |

Table 5. Descriptive and univariable logistic regression results for risk factors associated with heat related illness in dogs under primary veterinary care in the VetCompass^TM^ Programme in the UK during 2016.

| Independent Variable | Case No. (%) | Non-case No. (%) | Odds ratio | 95% CI | P-value |
| --- | --- | --- | --- | --- | --- |
| *Purebred* |  |  |  |  | < 0.001 |
| Non-designer Crossbred | 52 (13.33) | 193,878 (21.42) | Base |  |  |
| Designer Crossbred | 10 (2.56) | 52,019 (5.75) | 0.72 | 0.37-1.41 | 0.335 |
| Purebred | 326 (83.59) | 655,202 (72.39) | 1.86 | 1.38-2.49 | <0.001 |
| Unrecorded | 2 (0.51) | 4054 (0.45) | 1.84 | 0.45-7.55 | 0.398 |
| *Skull shape* |  |  |  |  | < 0.001 |
| Mesocephalic | 173 (44.36) | 452,295 (49.97) | Base |  |  |
| Dolicocephalic | 31 (7.95) | 75,817 (8.38) | 1.07 | 0.73-1.57 | 0.732 |
| Brachycephalic-cross | 1 (0.26) | 12,337 (1.36) | 0.21 | 0.03-1.51 | 0.122 |
| Brachycephalic | 131 (33.59) | 166,772 (18.42) | 2.05 | 1.64-2.58 | < 0.001 |
| Unknown | 54 (13.85) | 197,932 (21.86) | 0.71 | 0.53-0.97 | 0.03 |
| *Adult bodyweight (kg)* |  |  |  |  | < 0.001 |
| <10 | 61 (15.64) | 213,291 (23.56) | Base |  |  |
| 10-<20 | 90 (23.08) | 167,689 (18.53) | 1.88 | 1.36-2.60 | < 0.001 |
| 20-<30 | 76 (19.49) | 117,605 (12.99) | 2.26 | 1.61-3.17 | < 0.001 |
| 30-<40 | 37 (9.49) | 69,895 (7.72) | 1.85 | 1.23-2.79 | 0.003 |
| 40-<50 | 11 (2.82) | 19,848 (2.19) | 1.94 | 1.02-3.68 | 0.043 |
| ≥50 | 7 (1.79) | 6391 (0.71) | 3.83 | 1.75-8.38 | 0.001 |
| Unrecorded | 108 (27.69) | 310,434 (34.3) | 1.22 | 0.89-1.67 | 0.221 |
| *Bodyweight relative to breed/sex mean* |  |  |  |  | < 0.001 |
| Lower | 124 (31.79) | 317,225 (35.05) | Base |  |  |
| Equal/Higher | 156 (40.00) | 275,357 (30.42) | 1.45 | 1.15-1.84 | 0.002 |
| Unrecorded | 110 (28.21) | 312,571 (34.53) | 0.90 | 0.70-1.16 | 0.423 |
| *Sex/neuter* |  |  |  |  | 0.459 |
| Female-entire | 93 (23.85) | 233,734 (25.82) | Base |  |  |
| Female-neutered | 89 (22.82) | 197,792 (21.85) | 1.13 | 0.85-1.51 | 0.407 |
| Male-entire | 117 (30.00) | 259,405 (28.66) | 1.13 | 0.86-1.49 | 0.367 |
| Male-neutered | 91 (23.33) | 209,993 (23.20) | 1.09 | 0.82-1.45 | 0.563 |
| Unrecorded | 0 (0.00) | 4227 (0.47) | ~ | ~ | ~ |
| *Age* |  |  |  |  | 0.063 |
| <2 years | 78 (20.00) | 234,364 (25.89) | Base |  |  |
| 2-<4 years | 94 (24.10) | 178,135 (19.68) | 1.59 | 1.17-2.14 | 0.003 |
| 4-<6 years | 65 (16.67) | 139,916 (15.46) | 1.40 | 1.00-1.94 | 0.047 |
| 6-<8 years | 56 (14.36) | 113,325 (12.52) | 1.49 | 1.05-2.09 | 0.024 |
| 8-<10 years | 36 (9.23) | 90,982 (10.05) | 1.19 | 0.80-1.77 | 0.391 |
| 10-<12 years | 24 (6.15) | 66,241 (7.32) | 1.09 | 0.69-1.72 | 0.716 |
| ≥12 years | 34 (8.72) | 69,769 (7.71) | 1.46 | 0.98-2.19 | 0.064 |
| Unrecorded | 3 (0.77) | 12,421 (1.37) | 0.73 | 0.23-2.30 | 0.586 |
